# Stability Outcomes following Computer-Assisted ACL Reconstruction

**DOI:** 10.1155/2015/638635

**Published:** 2015-03-26

**Authors:** Melissa A. Christino, Bryan G. Vopat, Alexander Mayer, Andrew P. Matson, Steven E. Reinert, Robert M. Shalvoy

**Affiliations:** ^1^Division of Sports Medicine, Boston Children's Hospital, Boston, MA 02215, USA; ^2^Department of Orthopaedic Surgery, Massachusetts General Hospital, Boston, MA 02114, USA; ^3^Department of Orthopaedic Surgery, Rhode Island Hospital, Brown University, Providence, RI 02903, USA; ^4^Department of Orthopaedic Surgery, Duke University Medical Center, Durham, NC 27710, USA; ^5^Department of Information Services, Rhode Island Hospital, Lifespan, Providence, RI 02903, USA

## Abstract

*Purpose*. The purpose of this study was to determine whether intraoperative prereconstruction stability measurements and/or patient characteristics were associated with final knee stability after computer-assisted ACL reconstruction. *Methods*. This was a retrospective review of all patients who underwent computer-assisted single-bundle ACL reconstruction by a single surgeon. Prereconstruction intraoperative stability measurements were correlated with patient characteristics and postreconstruction stability measurements. 143 patients were included (87 male and 56 female). Average age was 29.8 years (SD ± 11.8). *Results*. Females were found to have significantly more pre- and postreconstruction internal rotation than males (*P* < 0.001 and *P* = 0.001, resp.). Patients with additional intra-articular injuries demonstrated more prereconstruction anterior instability than patients with isolated ACL tears (*P* < 0.001). After reconstruction, these patients also had higher residual anterior translation (*P* = 0.01). Among all patients with ACL reconstructions, the percent of correction of anterior translation was found to be significantly higher than the percent of correction for internal or external rotation (*P* < 0.001). *Conclusion*. Anterior translation was corrected the most using a single-bundle ACL reconstruction. Females had higher pre- and postoperative internal rotation. Patients with additional injuries had greater original anterior translation and less operative correction of anterior translation compared to patients with isolated ACL tears.

## 1. Introduction

Anterior cruciate ligament (ACL) reconstruction surgery is common, with approximately 125,000–175,000 procedures performed annually in the United States [1, 2]. Despite the large number of ACL reconstructions performed, the success rate of this procedure lags behind those of other common orthopedic procedures, and optimizing surgical technique to minimize failures has been the focus of the majority of ACL research.

Individual factors that have been associated with higher rates of failure after ACL reconstruction include younger age [3], higher activity level [4], female gender [5, 6], and ligamentous laxity [7, 8]. Additionally, injury factors such as mechanism of injury and concomitant lesions of the meniscus and articular cartilage have been shown to predict worse long term outcomes [9–11]. Historically, subclassification of ligamentous knee injuries has improved the accuracy of diagnoses and enabled treatments to be tailored toward specific injuries [12]. The fact that outcome has been shown to be associated with patient and injury specific factors suggests ACL injuries are not all the same and that patients may benefit from individualized treatment.

Computer navigation is increasingly being used in orthopaedic surgical procedures. In ACL reconstruction surgery, it has been shown to improve accuracy of bone tunnel placement [13–16]. It has also been shown to reliably obtain quantitative intraoperative measurements of knee stability [17, 18]. Many kinematic studies utilizing computer navigation have compared stability outcomes of single-bundle versus double-bundle techniques [19–26]. Others have described translational and rotational stability characteristics in cadaver and in vivo studies [27–29].

Few studies have specifically sought to define injury instability or have investigated the amount of translational and rotational correction that can be achieved using computer-assisted ACL reconstruction techniques. Evaluating the quantitative kinematics of the knee after injury as well as the changes that occur with reconstruction may provide valuable insight into the management of ACL injuries and could influence surgical decision making. Ohkawa et al. demonstrated that preoperative AP and rotational laxity varied among patients and suggested that postoperative stabilization may vary as well [29]. However, they did not stratify these differences by patient characteristics or additional injuries. The purpose of this study was to determine whether intraoperative prereconstruction stability measurements and/or patient characteristics were associated with final stability results after computer-assisted ACL reconstruction. It was hypothesized that preoperative rotational and translational stability would predict postoperative stability and that patient characteristics and concomitant intra-articular knee injuries would be associated with more pre- and postoperative instability.

## 2. Materials and Methods

This study was a retrospective review of all patients who underwent computer-assisted primary single-bundle ACL reconstruction by a single surgeon from 2007 to 2012. Exclusion criteria included revision surgeries and those patients with incomplete intraoperative data.

All patients had computer-navigated ACL reconstructions using the Aesculap 2.0 Ortho Pilot Navigation System (B. Braun Aesculap, Tuttlingen, Germany). Intraoperative pre- and post-ACL reconstruction stability measurements were collected; anterior translation, internal rotation, and external rotation were measured at 30 degrees of knee flexion (see Section 3).

Patient charts were reviewed for this intraoperative stability data as well as for relevant surgical details (graft type, fixation) as well as patient characteristics (age, gender, and associated injuries).

One hundred eighty-seven anterior cruciate ligament reconstructions were performed by a single surgeon between January 2007 and January 2012. Twenty-two were revision surgeries and were excluded from data analysis, and an additional 22 patients were excluded due to incomplete charts or documented problems with intraoperative stability measurements. Thus 143 patients with primary ACL reconstructions were included for analysis.

Preoperative rotational and translational stability was compared to postoperative stability, and stability data was compared between various patient and injury characteristics. Statistical analysis was performed using Pearson's correlation coefficients, *t*-tests, and ANOVAs. Significance was set at *P* < 0.05* a priori.*

## 3. Surgical Technique

All surgical ACL reconstructions were performed under general anesthesia with a tourniquet applied to the proximal thigh. The tourniquet was inflated for all patellar tendon graft reconstructions but not for hamstring or allograft (soft tissue) reconstructions. A diagnostic arthroscopy was performed with a 30-degree arthroscope. If present, all meniscal pathology was addressed with meniscal repair or partial meniscectomy prior to reconstruction. A modest notchplasty was performed if the notch was stenotic, and the ligament remnants were debrided to their footprints on the tibia and the femur.

For hamstring graft reconstructions, the gracilis and semitendinosus tendons were harvested prior to diagnostic arthroscopy, prepared, and doubled to create a quadrupled tendon graft construct. For patellar tendon graft reconstructions, the tourniquet was inflated after diagnostic arthroscopy and a 10 mm central strip of tendon harvested. A 20 mm bone plug was harvested from the inferior pole of the patella in line with the tendon graft and a similar 25 mm bone plug harvested from the tibial tubercle.

**Figure 1:**
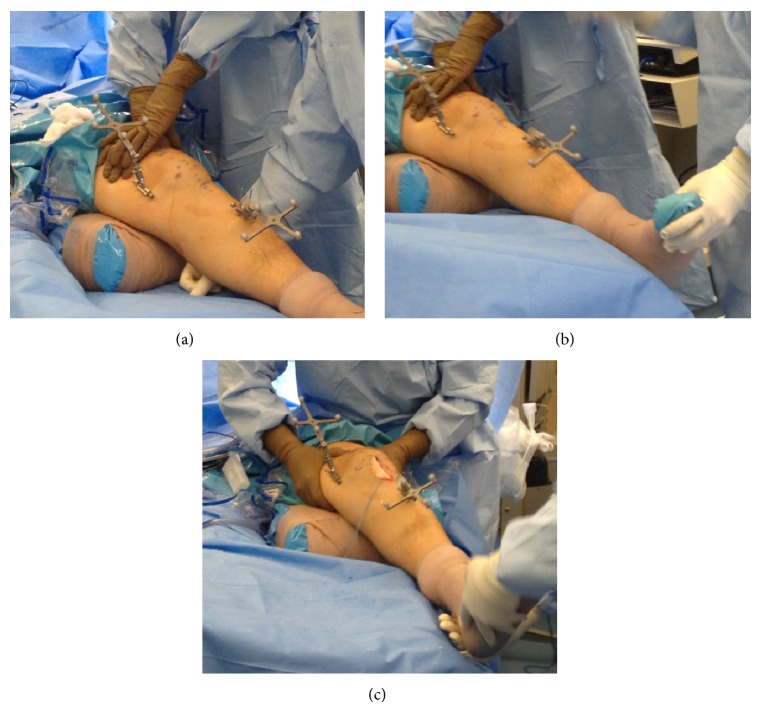
Intraoperative photographs showing positioning of the femoral and tibial navigation transmitters and demonstrating the stability testing maneuvers: anterior translation (a), internal rotation (b), and external rotation (c).

For all surgeries, the 2.0 Ortho Pilot (B. Braun Aesculap, Tuttlingen, Germany) Computer Navigation System was used to calculate knee kinematics before and after reconstruction. Tibial and femoral transmitters were applied with two 2.5 smooth K-wires each (Figure 1). Intra-articular and extra-articular landmarks were registered and kinematic acquisition was achieved by ranging the knee from 0 to 90 degrees of flexion. The knee was secured manually on a semirigid bolster with the knee at 30 degrees of flexion, which is the standard position for the Lachman examination of the ACL. Stability testing was performed by the senior surgeon in all cases. Maximum manual AP stress was applied to the posterior calf for three trials (Figure 1(a)), and the resulting values of AP translation, external rotation, and internal rotation were recorded for this maneuver. Maximum manual internal rotation (IR) was then applied to the foot for three trials (Figure 1(b)), and associated values of AP translation, external, and internal rotation were recorded. Maximum manual external rotation (ER) was also applied to the foot for three trials, and corresponding AP translation, external rotation, and internal rotation were again recorded (Figure 1(c)).

Computer navigation and arthroscopic visualization were used to identify anatomic tibial and femoral tunnel placement and avoid graft impingement. Soft tissue grafts were fixed by 9-10 Intrafix devices (DePuy Mitek, Raynham, MA) on the femur and 8–10 Intrafix (Depuy Mitek, Raynham, MA) devices on the tibia. Patellar tendon grafts were fixed with Biosure HA interference screws (Smith & Nephew, Andover, MA) on the femur and tibia.

With graft fixation completed, stability testing was repeated and documented in the same manner as previously described.

## 4. Results

A summary of patient demographics, injury characteristics, and type of ACL reconstructions can be seen in Table 1. The five most common mechanisms of injury were soccer (23), nonspecified sports (18), nontraumatic injuries (18) such as falls or twists while standing, skiing (14), and basketball (14).

The average pre- and postreconstruction anterior drawer, internal rotation, external rotation, and the percent of correction for each of these values can be seen in Table 2. There were no significant correlations found between prereconstruction rotation measurements and either the pre- or postreconstruction anterior drawer. When total rotation was calculated by adding maximum external and internal rotation, total rotation had a weak but significant correlation with prereconstruction anterior drawer (*r* = 0.27, *P* = 0.001).

The average percent of correction between pre- and postreconstruction anterior drawer was significantly higher than the percent of correction for pre- and postreconstruction internal rotation (*P* < 0.001) and external rotation (*P* < 0.001). The percent of correction for internal rotation was also significantly higher than the percent of external rotation correction (*P* < 0.001). Postreconstruction total rotation was found to be significantly less than prereconstruction total rotation (29.3 degrees versus 38.9 degrees, *P* < 0.001).

When examining stability data by gender, there were no statistically significant differences in prereconstruction anterior drawer or external rotation, but females had significantly more prereconstruction internal rotation than males (24.1 degrees versus 20.4 degrees, *P* < 0.001). Females were also found to have significantly higher total rotation numbers before reconstruction when compared to males (41.3 degrees versus 37.4 degrees, *P* < 0.001). Looking at postreconstruction measurements, females continued to have significantly higher internal rotation values than males (16.8 degrees versus 13.7 degrees, *P* = 0.001) as well as total rotation (30.9 degrees versus 28.2 degrees, *P* = 0.002).  Table 3 summarizes these results.

Seventy-one patients (49.7%) had additional intra-articular injuries in addition to an ACL tear, and these included meniscal tears, chondral injuries, and capsular tears. Sixty-six (92.3%) of these patients had at least 1 meniscal tear, and the breakdown of injuries can be seen in Table 4. Patients with additional intra-articular injuries showed more anterior instability before reconstruction than patients with isolated ACL tears (15.7 mm versus 13.3 mm, *P* < 0.001), although there were no significant differences in rotation. After reconstruction, patients with additional injuries had statistically higher residual anterior drawer measurements than those with isolated ACL tears (5.2 mm versus 4.4 mm, *P* = 0.01).

There were no significant differences in postreconstruction anterior translation or rotational stability measurements among the different graft types used.

## 5. Discussion

This is the largest series reporting intraoperative knee stability following ACL reconstruction and comparing patient and injury characteristics with computer-navigated stability data. Our study found that anterior translation was corrected more than rotation using a single-bundle reconstruction. Women were also found to have more pre- and residual postreconstruction internal rotation than men, and patients with additional intra-articular injuries had more anterior laxity both before and after reconstruction. These results suggest that subtleties exist among ACL injuries and future research needs to be done to account for individual variability related to rotation, gender, and associated injuries.

Using computer navigation for ACL reconstructions has not been shown to improve knee stability or functional outcomes in patients compared to conventional ACL reconstructions [30]. However, the value of computer navigation may lie in the ability to define and identify subtleties among injuries. Quantitatively defining injury characteristics of ACL tears and their subsequent repairs may play an important role in guiding treatment and surgical decision making. Reporting these findings may be an important first step in trying to stratify differences seen in patients and injury patterns immediately affecting pre- and postoperative stability.

Ohkawa et al. demonstrated that preoperative AP and rotational laxity varied among patients and suggested that postoperative stabilization may vary as well [29]. However, they did not stratify these differences by patient characteristics or additional injuries. Our study demonstrated that females had greater internal rotation and patients with additional intra-articular injuries had greater anterior laxity. The clinical significance of these findings has yet to be elucidated, but these patients may be considered for adjunct procedures or perhaps double-bundle reconstruction techniques, which have been proposed to control for rotation more than single-bundle reconstructions [31–33].

A recent retrospective review of 55 patients with computer-navigated ACL reconstructions found that double-bundle reconstruction had significantly greater rotational stability than single-bundle reconstructions [34]. However, female patients who had computer-navigated double-bundle ACL reconstructions had significantly worse outcome scores at 2 years than males. In this study, they also reported higher preoperative internal rotation values for females who had either single- or double-bundle reconstructions compared to males but found no difference postoperatively. This is similar to our study; however we also found increased internal rotation in women immediately postoperatively using a single-bundle reconstruction. Females have been found to have more ligamentous laxity in general when compared to males [8], and the rotational stability differences identified in this study support the idea that unique anatomic and physiologic differences may exist between sexes, and they should be taken into account with surgical decision making and management of ACL injuries.

Similarly, our study suggests that isolated ACL tears may behave differently than tears with associated additional intra-articular pathology. We showed that patients with at least one additional intra-articular injury had higher pre- as well as postreconstruction anterior translation compared to isolated ACL tears. Logically, a more severe injury would be more unstable. The menisci, capsular structures, and collateral ligaments all contribute to providing inherent stability to the knee, so it is not surprising that if these are also injured, the knee may be more unstable initially and more difficult to stabilize. Particularly if injured meniscal tissue needs to be resected with partial meniscectomy, as was the case for most of our meniscal tears, this may negatively contribute to final stability of the knee. More significant injuries may require more rigorous stabilization techniques, but the intricacies of complex injury patterns need to be further evaluated for their clinical significance and impact on the overall stability of the knee during ACL reconstruction.

There were limitations to this study. First, the study was retrospective in nature. Second, some patients had to be excluded due to incomplete medical records, and their data was thus not included in the analysis. Third, while surgical technique was performed by the same surgeon in a systematic standardized way, the possibility exists for error in obtaining navigation measurements as manual stress was applied in conjunction with automated measurements. Last, only intraoperative knee stability was assessed, and initial stability may not predict the final stability after healing and in the clinical setting [29]. However, our focus was to define initial injury and reconstruction characteristics using computer navigation. Follow-up evaluation of knee stability and outcome measures were beyond the scope of the current study, but they should be evaluated in the future.

## 6. Conclusions

Anterior translation was found to have the most correction using a single-bundle ACL reconstruction. Females were found to have higher pre- and postoperative internal rotation. Patients with additional intra-articular knee injuries had greater original anterior translation and less operative correction of anterior translation when compared to patients with isolated ACL tears.

## Figures and Tables

**Table 1:** Patient demographics, injury characteristics, and type of ACL reconstructions.

Patient characteristics	
Total number of patients	143
Gender	
Male	87 (60.8%)
Female	56 (39.2%)
Average age in years (SD)	29.8 (±11.8)

Injury characteristics	
Knee affected:	
Right	71 (49.7%)
Left	72 (50.3%)
Isolated ACL tear	72 (50.3%)
ACL tear with additional intra-articular injuries	71 (49.7%)

Type of ACL reconstruction	
Hamstring autograft	63 (44.1%)
Patellar tendon autograft	54 (37.8%)
Allograft	24 (16.8%)
Combination of hamstring autograft with allograft augmentation	2 (1.4%)

**Table 2:** Pre- and postreconstruction stability measurements.

	Prereconstruction	Postreconstruction	Percent of correction
Anterior drawer mm (SD)	14.47 (±3.41)	4.80 (±2.05)	65.7% (±15.4%)

Internal rotation degrees (SD)	21.86 (±4.37)	14.99 (±4.39)	31.3% (±17.1%)

External rotation degrees (SD)	17.08 (±3.80)	14.29 (±3.52)	15.2% (±19.2%)

**Table 3:** Gender differences pre- and postreconstruction.

	Prereconstruction		Postreconstruction	
	Male	Female	Male	Female
Anterior drawer mm (SD)	14.47 (±3.01)	14.4 (±3.67)	5.02 (±2.15)	4.45 (±1.86)

Internal rotation degrees (SD)	^*^20.45 (±4.15)	^*^24.05 (±3.79)	^*^13.86 (±4.2)	^*^16.75 (±4.11)

External rotation degrees (SD)	17 (±4.09)	17.21 (±3.34)	14.39 (±3.21)	14.13 (±3.97)

Total rotation degrees (SD)	^*^37.45 (±5.2)	^*^41.27 (±4.77)	^*^28.25 (±4.6)	^*^30.89 (±5.49)

^*^denotes significance with *P* < 0.05.

**Table 4:** Distribution of additional intra-articular injuries.

Additional intra-articular injuries (*n* = 71)	Number	Percentage
Isolated medial meniscus tear	27	38.0%
Isolated lateral meniscus tear	25	35.2%
Combined medial and lateral meniscal tears	8	11.3%
MCL tear (2 also with medial capsular tears)	4	5.6%
Medial meniscus tear with chondral injury	2	2.8%
Lateral meniscal tear with chondral injury	1	1.4%
Lateral meniscal tear with medial capsular tear	1	1.4%
Combined medial and lateral meniscal tears with chondral injury	1	1.4%
Combined medial and lateral meniscal tears with medial capsular injury	1	1.4%
Isolated medial capsular tear	1	1.4%
